# A Mixed Methods Study to Examine the Influence of CLIL on Physical Education Lessons: Analysis of Social Interactions and Physical Activity Levels

**DOI:** 10.3389/fpsyg.2020.00578

**Published:** 2020-03-27

**Authors:** Celina Salvador-García, Carlos Capella-Peris, Oscar Chiva-Bartoll, Pedro Jesús Ruiz-Montero

**Affiliations:** ^1^Faculty of Education, International University of La Rioja, Logroño, Spain; ^2^Department of Education and Specific Didactics, Jaume I University, Castellón de la Plana, Spain; ^3^Department of Physical and Sports Education, University of Valencia, Valencia, Spain; ^4^Faculty of Education and Sport Sciences, University of Granada, Melilla, Spain

**Keywords:** physical education, CLIL, moderate-vigorous physical activity, pedagogical approach, mixed methods, social relationships

## Abstract

Physical Education is often selected for applying multilingual initiatives through the use of a content and language integrated learning (CLIL) approach. However, it is still unclear whether the introduction of such an approach might entail losing the essence of physical education and distorting its basic purposes. The aim of this study is to analyze the impact of CLIL on physical education lessons. Given the purpose of this study, a mixed methodological approach based on a sequential exploratory design divided into two different phases is used. We begin with initial qualitative data collection (phase I), consisting of the analysis of interviews with 12 participants (8 teachers and 4 students). Based on its analysis, two foci are identified: social relationships and physical activity. Then, informed by the results obtained, a quantitative approach is used (phase II), differentiating these two sets of data to make a more in-depth analysis of them. On the one hand, a sociometric questionnaire was applied to analyze the social relationships between CLIL students. On the other hand, a quasi-experimental design (*n* = 49) was implemented using accelerometry to measure moderate to vigorous physical activity (MVPA) in the physical education sessions. Regarding physical activity, the results show that levels of MVPA are higher in the experimental group (CLIL) than in the control group, a result which clarifies the divergent viewpoints of the interviewees. However, focusing on social relationships, the sociometric questionnaire results show that there were no statistically significative changes, although some signs of a slight effect on students’ relationships arise depending on their gender. Therefore, more research would be necessary to further study the effect of CLIL in this regard.

## Introduction

Currently, Physical Education has been claimed to generate different ‘educationally beneficial outcomes for students, across a range of domains’ (Kirk, 2013, p. 978). This may be explained by the fact that this subject is defined by the two words that form its name (Kirk, 2010). In other words, Physical Education has the potential to promote learning related to different spheres, not only the physical one, but also the social, personal and cognitive (Dyson et al., 2004; McEvoy et al., 2017). However, there are various historical and philosophical accounts outlining the journey Physical Education has taken throughout its history, and even today there may be different viewpoints on its current understanding. As an example, some purposes of Physical Education may be: (1) development of motor and sport-specific skills, (2) promotion of health-related fitness and active lifestyles, and (3) personal, social and moral development (Hardman, 2011).

Despite Physical Education’s holistic power, there is a ‘growing movement to develop and adopt classroom-based physical activity in an effort to increase physical activity within the school day’ (Quarmby et al., 2019, p. 2). This tendency is related to the aforementioned first and second functions of Physical Education, and responds to the worrisome concern due to the decrease in moderate to vigorous physical activity (MVPA) in school-aged children (Hollis et al., 2017; Viciana et al., 2019). In fact, Physical Education lessons are the only opportunity to engage in physical activity for many adolescents and children (Meyer et al., 2013; Aljuhani and Sandercock, 2019). Therefore, this subject is crucial in contributing to the recommendations on daily amount of physical activity (World Health Organization, 2010; Viciana et al., 2019).

The third purpose of Physical Education stated by Hardman (2011) refers to personal, social and moral development; and it usually constitutes one of the main goals of European Physical Education programs (Opstoel et al., 2019). Indeed, Physical Education is seen as a great opportunity to develop the students’ personality and socialization (Weiss, 2011; Andueza and Lavega, 2017) given its social nature and the particular context it generates. However, it is important to mention that simply participating in Physical Education lessons does not automatically lead to the development of such skills (Cryan and Martinek, 2017; Opstoel et al., 2019).

In addition to the aforementioned functions, Physical Education has increasingly been involved in the development of programs devoted to the learning of another language (Baena-Extremera et al., 2017; Lamb and King, 2019), often through the CLIL (Content and Language Integrated Learning) approach (Coral et al., 2017; Salvador-García and Chiva-Bartoll, 2017). This stems from the need to improve second-language education and bilingualism, which is an essential skill in today’s society (Marsh, 2002). However, several voices claim that non-linguistic subjects, as is the case of Physical Education, are undervalued when this approach is applied (Pérez-Cañado, 2017). Furthermore, the introduction of CLIL inevitably entails a change in the development of the lessons (Coyle, 2015). Consequently, it is worrisome that Physical Education might lose its identity when introducing another language in class (Merino, 2016), because this subject should ensure that its particular purposes are achieved.

The main aim of this study is to analyze the impact of CLIL on Physical Education lessons, bearing in mind the essential functions of the subject.

## Mixed Methods

All methods of data collection have limitations. Qualitative methods can provide in-depth information whereas quantitative methods can test predictive associations. Mixed-methods studies collect and combine qualitative and quantitative data in order to build on the complementary strengths of both qualitative and quantitative methods (Creswell and Plano Clark, 2017). In this regard, a considerable amount of literature defends that mixing different types of methods can strengthen a study (Greene and Caracelli, 1997).

In order to utilize the benefits of each method, we employed a sequential exploratory design to integrate qualitative and quantitative methods (Creswell and Plano Clark, 2017). This design was conducted in two phases, with equal status given to both phases of research. The qualitative data collected in Phase 1 informed the design of the quantitative study in Phase II. Such a design was chosen because there was a need to begin with initial qualitative data collection so as to identify the focus of the possible variables to examine (Tashakkori and Teddlie, 1998). In this case, we needed to narrow the possible effects of CLIL on Physical Education in order to analyze them objectively.

**FIGURE 1:**
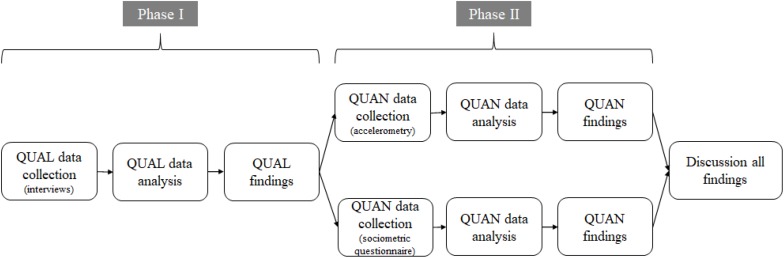
Stages of the sequential exploratory design.

Participants in the first phase of data collection were high school students and teachers who had been using the CLIL approach in Physical Education lessons. From the interviews, we identified two main themes related to the influence of CLIL on Physical Education as a subject. Finding out whether there was an effect due to CLIL on these two themes became the two branches investigated in phase II of the study. In this second phase, we used a sociometric questionnaire and accelerometry to test the predictive associations identified in the interviews. This quantitative approach allowed us to examine whether the qualitative findings were supported by objective measures. Both approaches carried equal weight in the resulting discussion. Figure 1 shows the stages followed in the design. The following sections detail each phase, including participants, method, data analysis and findings of each.

### Phase I (Qualitative Study)

#### Research Question

What impact will CLIL have on Physical Education lessons from the perspective of the students and teachers involved?

#### Participants

On the one hand, eight Spanish teachers of Physical Education with CLIL (four female and four male) composed the sample of the study. They are teachers in different high schools and they have been implementing the CLIL approach for at least 3 years. Furthermore, they all have an official certificate that enables them to carry out Physical Education lessons in English. On the other hand, four 15- to 17-year-old Spanish students were also selected. In their case, they had been doing Physical Education through CLIL for at least a term in their high school.

Intentional sampling was chosen in an attempt to achieve representativeness among both teachers and students (Patton, 2002). Therefore, regarding the teachers, we considered features such as age (25–40 *n* = 4; 41–60 *n* = 4), gender (female *n* = 2; male *n* = 6) and CLIL experience (3–5 years *n* = 4; 5–8 years *n* = 4). With respect to the students, the representative quotas of participants were based on their gender (female *n* = 2; male *n* = 2), English marks for the last academic year (high marks *n* = 2; low marks *n* = 2) and Physical Education marks for the last academic year (high marks *n* = 2; low marks *n* = 2).

All the participants had received detailed information about ethical considerations regarding informed consent and confidentiality, building on guidelines of the ethics committee of Universitat Jaume I and had thereafter agreed to participate in the study. In addition, the students’ parents or guardians signed an informed consent document.

#### Method

All participant interviews were carried out by the same interviewer. We used a semi-structured interview format because, according to literature, this instrument allows the participants to describe detailed personal information, but the interviewer still has good control over the information received. In addition, they ensured a degree of comparability across interviews and allowed for different themes to arise (Creswell, 2012). Therefore, the use of this type of data collection was appropriate to identify the possible effects of CLIL on Physical Education, which was the main topic of the interview guide. Previous literature helped us to frame the interview questions, which were organized around the aims of Physical Education (Hardman, 2011), although other questions emerged from the dialog between interviewer and interviewees to probe for more relevant data (Mackey and Gass, 2005; DiCicco-Bloom and Crabtree, 2006). The interviews started with general questions, followed by other more specific ones to reconstruct the interviewees’ subjective theories without biasing them (Flick, 2014). The structure of the interviews is presented in Table 1.

**TABLE 1:** Structure of the interviews.

	Issues addressed			
	Teachers’ interviews		Students’ interviews	
Interview parts	Issues	Basic interview guide	Issues	Basic interview guide
“Ice-breaker” questions	Information on personal matters, language proficiency, work experience and educational, and training attainments.	What is your teaching experience (and using CLIL)? What is your English level? What specific training have you undergone to do CLIL?	Information on personal matters, general opinions about the subject.	• What do you think of Physical Education? • How do you feel about the subject?
General questions	Preparation of the classes.	• What is your general opinion regarding Physical Education with CLIL? • How do you prepare your CLIL lessons?	Learning in physical education The acquisition of language skills.	• Has CLIL hindered your learning of the subject? How? Why (not)? • Why was CLIL (not) helpful for language learning?
Specific questions	Class development: strengths, limitations. Differences between using and not using the CLIL approach.	• What are the advantages of using CLIL in Physical Education? • What are the disadvantages of using CLIL in Physical Education? • What are the differences in CLIL and non-CLIL Physical Education? And regarding motor and sport-specific skills/activity or movement/personal, social and moral development?	Advantages and disadvantages of the CLIL approach in physical education classes. Differences regarding classes without the CLIL approach.	• Why can CLIL be beneficial for students in Physical Education? • What are the disadvantages of using CLIL in Physical Education? • What are the differences in CLIL and non-CLIL Physical Education? And regarding motor and sport-specific skills/activity or movement/personal, social and moral development?
Conclusion question	Further observations			

The initial part of the interview involves the interviewer making a brief presentation, explaining to the interviewees the following points: the confidentiality of their identity, the use of recording exclusively for research purposes, the possibility of stopping the recording at any time during the interview and the non-obligation to answer all questions. They also clarified that the interviews were anonymous, as recommended by the protocols of this technique.

The teachers’ interviews were individual, whereas the students were interviewed as a group to combine individual experiences and build a collective discourse, comparing and contrasting different viewpoints and therefore avoiding any distortion of their perceptions. All the interviews were recorded with a SONY ICD-P530F recorder and they were carried out in Spanish. After the data analysis a professional translator translated them into English for publication. Pseudonyms are used to protect the interviewees’ identities.

#### Data Analysis

Initially, the research team read all transcripts to provide a holistic review of the participants’ viewpoints on the effects of CLIL on Physical Education. Next, we exported the interviews into NVivo-11 software. Later, we met to discuss initial reactions and develop potential categories related to the essential features of Physical Education. Then, the representative quotes were grouped together within these categories. Finally, a member checking process was carried out to ensure inter-rater reliability (Johnson and Christensen, 2016).

#### Findings

The research team identified two main categories that pointed to the effects generated by CLIL on Physical Education according to the interviewees’ viewpoints. In this respect, ‘social relationships’ and ‘physical activity’ were the aspects that could be affected by the use of this innovative approach.

##### Social relationships

With regard to the ‘social relationships’ category, the idea that CLIL entails a change was shared by all interviewees. For example, the students clearly stated that ‘*speaking in English in the lessons helped us to be more attentive with our classmates. If you were able to understand the instructions, you were actively involved in helping the rest of the students*’ (Student 2). Consequently, students were led to increase their work as a team or ‘*group collaboration: since I don’t understand, you explain it to me*’ (Student 3).

The teachers noticed an increase in the students’ helpfulness too, as is illustrated in the following quote:

‘*There is more collaboration among students, of course. I think that any handicap you (the teacher) bring to class will be a challenge for the pupils and, therefore, it will help (to enhance teamwork). In this case, it was English language, but (*…*) any new handicap will lead to an increase in everyone’s collaboration*’ (Teacher 3).

In addition, according to the teachers, this increase in students’ interactions was also a consequence of the type of tasks and the methodology that CLIL entails.

‘*One of the ways of promoting language use is by doing cooperative tasks. When planning the sessions, there is a need to search for cooperative structures to boost language use. In addition, I can help because it is something mandatory at a specific moment of the lesson (*…*) but I believe that showing a weakness and sharing it with your classmates, being conscious of the fact that we are more similar than we had thought, being aware of our sameness*… *Sharing all these feelings generates an emotional atmosphere that truly promotes learning with the other*’ (Teacher 4).

All in all, according to the interviewees’ perceptions, the use of CLIL increases collaboration and the feeling of sameness within the group while enhancing the number of interactions between students. Therefore, CLIL might entail a change in the social atmosphere of the group.

##### Physical activity

Physical activity was the second category identified. However, participants reported divergent perspectives regarding this aspect. On the one hand, all students share a common discourse, which is also supported by some of the Physical Education teachers. According to their viewpoint, the implementation of CLIL does not hinder the amount of physical activity carried out in Physical Education lessons. The following quotes illustrate this idea:

‘*I have adapted the theoretical content, but the practical content has not been adapted*’ (Teacher 2).

‘*The activities and the practice itself have remained the same*’ (Teacher 7).

In the same vein, despite the required methodological changes due to CLIL that were not noticed by students, one of them stated that ‘*it is exactly the same learning in English or in Spanish. The only difference is that we use a different language*’ (Student 1). In fact, none of the adolescents thought that CLIL could diminish their time engaged in physical activity.

On the other hand, some teachers were concerned with this issue and showed an opposing opinion. Specifically, they believed that the use of a different language implied more teacher talking time and a slowing of students’ understanding that could also decrease the students’ physical activity time. In the words of one of the teachers:

‘*Obviously, the explanations are longer. You have to explain the same thing (compared to a non-CLIL group) and you (the teacher) do not have the same knowledge, skill and speed when speaking in English*’ (Teacher 3).

Also, a different teacher reported that although physical activity was still an important part of the lessons, the amount of time that students were engaged in it could be affected because it was more difficult for students to understand an activity and start it. This can be perceived in the following quote, in which the teacher mentions both aspects:

‘*I think that the sport’s essence, its physical activity, was the same, it (CLIL) was not an obstacle. They may be slower to understand an activity, but once you have performed it, students worked with no difference (compared to non-CLIL students)*’ (Teacher 4).

To sum up, despite the fact that all interviewees thought that students’ quality of movement was the same regardless of CLIL, some teachers did worry about the amount of time that students were engaged in physical activity.

### Phase II (Quantitative Study)

#### Hypotheses

– The social relationships measured by the sociometric questionnaire conducted by the experimental group will improve after application of the CLIL approach.
– The amount of time that students are engaged in physical activity will be less for the experimental (CLIL) group than for the control (non-CLIL) group.

#### Participants

Once the findings of the first phase of the study were clear and the foci of the subsequent phase of the study were established (social relationships and physical activity), the participants for the second phase were recruited. In total, the convenience sample for the quantitative study consisted of 49 13- to 14-year-old students. Participants in the accelerometry analysis were divided into the experimental group (CLIL), composed of 13 girls and 10 boys, and the control group (non-CLIL), composed of 19 girls and 7 boys. This sample presented a statistically proportional distribution in terms of the gender variable between the groups χ^2^(*N* = 49) = 1.05, *p* = 0.306. The mean age was 13.8 (±0.18). Only the experimental group participated in the sociometric questionnaire measures. They did not have prior experience in Physical Education through CLIL. We decided that the group of participants in phase II of the study would not be the same as in phase I in order to avoid their attitudes and behavior being influenced by the interviews carried out in advance. Therefore, a different group of students, without CLIL experience, was selected.

For this part of the study the principal and the Physical Education teacher agreed to participate by establishing a memorandum of understanding. The students’ parents or guardians also signed an informed consent document.

#### Measures

To identify possible changes in the social relationships of the students in the experimental group, a sociometric questionnaire was applied at the beginning and at the end of the term. The three units included within this period were the first time that the students in the experimental group had done Physical Education through the CLIL approach. Sociometric methods are the most common form of measuring the status of individuals within groups to understand their relationships (Chelcea, 2005). The sociometric questionnaire consisted of four questions where students were asked to express sympathetic relationships in terms of their attraction or repulsion to classmates. These questions were: (a) Which classmates do you most like being with in Physical Education lessons? (b) Which classmates do you least like being with in Physical Education lessons? (c) Which classmates can help you to learn more in Physical Education lessons? (d) Which classmates can’t help you to learn in Physical Education lessons? A peer nomination technique in which participants were asked to nominate peers in order of importance was used. It is imperative to note that pre-test measures were taken before the first CLIL unit started. In addition, due to the sensitive nature of the questionnaire, students were provided with a private area to individually complete them and asked not to discuss the task with their classmates.

In order to discern whether there was an effect on students’ physical activity levels, the instruments selected were accelerometers (a device that measures the acceleration of a body through high-frequency recordings in order to discriminate behavioral patterns of physical activity). They are considered one of the most reliable ways of measuring levels of physical activity and were selected because they do not interfere with Physical Education sessions (Calahorro et al., 2015). In this study, the GENEActiv Original triaxial accelerometer was used. Data recruitment took place during a unit taught in January–February 2017. Both the experimental and control group had two 50-min lessons per week in the morning, in which accelerometer-based outcome measures were taken from all the students. The research design corresponds to a quasi-experimental design based on non-equivalent natural groups to make an objective analysis of physical activity levels in Physical Education lessons. The same Physical Education teacher taught both groups the same content (athletics) in an attempt to control potential ‘teacher-related’ confounding. Each student wore an accelerometer for the entire lesson on his/her left wrist. To control differing durations of Physical Education lessons, physical activity data were collected during the entire lesson because the English language was used with the experimental group throughout this time. The measurements were taken in six practical sessions of each group.

An experienced external observer conducted practice observations of all the lessons of both groups and completed the CLIL planning and observation checklist (Mehisto et al., 2008) to ensure this approach was properly implemented.

#### Data Analysis

Regarding the sociometric questionnaire, the data were analyzed to produce descriptive categorical results. The analysis of social relationships was conducted using the student peer nomination questionnaire with the experimental group (CLIL) in Spanish. After the peer nomination questionnaires were collected, frequency of positive and negative nominations was calculated for each student to determine social dynamics within the group. The mean scores of the ratings were computed for each student. Specifically, the number of nominations was divided by the number of students who took the questionnaire. This analysis let us identify the increase, maintenance or decrease in the number of positive and negative nominations.

For its part, we followed previous literature on accelerometry and we conducted the analyses unifying the moderate and vigorous categories into one variable called Moderate-Vigorous Physical Activity (MVPA). Specifically, the time spent on MVPA was calculated by applying previously calibrated and validated cut-off points for this type of population (Phillips et al., 2013). The data recorded were exported into the statistical package SPSS-24. Then, a multivariate analysis was used to analyze the effect of the group variable on MVPA time.

#### Findings

With reference to the sociometric questionnaire, a *t*-student analysis was conducted to determine whether there were differences between pre- and post-test measures regarding the four questions about relationships with classmates. This analysis was also performed differentiating by gender. The results do not show significant differences between the students’ score pre- and post-test measures. However, Table 2 also presents some interesting outcomes when comparing the results obtained by males and females. Regarding question (c) “Which classmates can help you to learn more in Physical Education lessons?”, girls increased their scores from pre-test (*M* = 0.96, *SD* = 0.113) to post-test (*M* = 0.141, *SD* = 0.183), whereas boys decreased their scores from pre-test (*M* = 0.134, *SD* = 0.226) to post-test (*M* = 0.061, *SD* = 0.182). In fact, only one of the questions, (b), shows similar trends between boys and girls. These results suggest that the CLIL program may have different effects on students’ relationships depending on their gender.

**TABLE 2:** Difference between study samples by gender and total in four sociometric questions about sympathetic relationships in terms of attraction or repulsion to classmates.

	Female group (*n* = 13)				Male group (*n* = 10)				Total group (*n* = 23)			
Sociometric questions	Pre-intervention	Post-intervention	*p*-value	Cohen’s *d*	Pre-intervention	Post-intervention	*p*-value	Cohen’s *d*	Pre-intervention	Post-intervention	*p*-value	Cohen’s *d*
	Mean (*SD*)	Mean (*SD*)			Mean (*SD*)	Mean (*SD*)			Mean (*SD*)	Mean (*SD*)		
(a) …classmates you most like in PE…	0.117 ± 0.189	0.121 ± 0.056	0.893	−0.028*	0.131 ± 0.178	0.121 ± 0.160	0.726	0.059*	0.122 ± 0.071	0.121 ± 0.061	0.912	0.003
(b) …classmates you like least in PE…	0.118 ± 0.139	0.134 ± 0.182	0.497	−0.098	0.108 ± 0.161	0.117 ± 0.087	0.770	–0.069	0.114 ± 0.107	0.126 ± 0.145	0.470	–0.094
(c) …classmates who can help you learn in PE…	0.096 ± 0.133	0.141 ± 0.183	0.582	−0.238*	0.134 ± 0.226	0.061 ± 0.182	0.396	0.355*	0.113 ± 0.176	0.105 ± 0.149	0.895	0.049
(d) …classmates who can’t help you learn in PE…	0.123 ± 0.132	0.143 ± 0.216	0.821	−0.111*	0.132 ± 0.195	0.086 ± 0.112	0.609	0.289*	0.126 ± 0.158	0.119 ± 0.175	0.900	0.041

*Opposing trends depending on gender.

For its part, the multivariate analysis performed showed significant main effects for the group variable [Wilks’ Λ = 0.637, *F*(1,49) = 25.664; *p* < 0.001; η^2^ = 0.363] on students’ physical activity level. In addition, partial eta square showed that the differences between the score obtained by the control group (non-CLIL) and the experimental group (CLIL) in students’ physical activity levels (sedentary-light and moderate-vigorous) is 36.3%. Table 3 presents the mean percentages of time that both groups spent at levels of sedentary-light and moderate-vigorous physical activity throughout the unit.

**TABLE 3:** ANOVA for students’ physical activity level (SLPA/MVPA) by group of participants (CLIL/non-CLIL).

Physical activity level	Group	Mean (*SD*)	*F*	η^2^
SLPA	Non-CLIL	72.79 (4.28)	25.664***	0.363
	CLIL	65.95 (4.95)		
MVPA	Non-CLIL	27.21 (4.28)	25.664***	0.363
	CLIL	34.04 (4.95)		

***p < 0.001. *Mean refers to percentage per session. SLPA, sedentary-light physical activity; MVPA, moderate vigorous physical activity*.

## Discussion of Qualitative and Quantitative Findings

The integrated mixed methods research strategy with a two-phase exploratory sequential design has been instrumental in improving our understanding of CLIL in the Physical Education field to examine the influence of this approach on the subject’s basic purposes: (1) development of motor and sport-specific skills, (2) promotion of health-related fitness and active lifestyles, and (3) personal, social and moral development (Hardman, 2011). While directly administered interviews in the first phase have been useful to narrow the study’s aims, the use of a sociometric questionnaire and accelerometry in the second phase allowed us to analyze social relationships and physical activity levels objectively within Physical Education lessons.

Despite the fact that mixed approaches are increasingly being advocated as a means to better comprehend educational concepts and contexts (Johnson and Onwuegbuzie, 2004; Tolan and Deutsch, 2015), there are still limited examples of their combination in the literature. Combining qualitative and quantitative methods enhanced the validity of our findings because we could triangulate results that examine the same phenomenon across different methods, we could expand and better elaborate our findings, and we could unveil contradictory findings that resulted from the use of different methods (Greene et al., 1989; Tolan and Deutsch, 2015). Relevant findings emerged at each phase of the research process and are reported by the two foci into which phase II is divided.

Consistent with previous literature, participants described how Physical Education with CLIL provided students with more options to help one another, interact with other peers, be more attentive, increase teamwork and share similar weaknesses (Coyle et al., 2010; Coyle, 2015; Salvador-García et al., 2018; Lamb and King, 2019). These aspects are linked to the personal and social spheres of Physical Education (Dyson et al., 2004; Hardman, 2011). Their mention by the interviewees may be explained because CLIL entails a type of talking and interaction that is different from that of traditional lessons (Casal, 2016), resulting in the promotion of social skills and peer relations. On this account, CLIL programs go beyond the mere usage of the target language in content in order to include other essential lifelong skills such as social ones (Torres-Rincon and Cuesta-Medina, 2019).

The interviews illustrate that the Physical Education teachers interviewed feel the need to use collaborative activities when planning lessons through the CLIL approach. In this regard, the introduction of collaborative activities can also be useful to strengthen social dynamics within the group, enhancing prosocial behavior and empathy among students (Andueza and Lavega, 2017). In addition, this means that CLIL may entail a change in Physical Education methodology, making it more participative, inclusive and collaborative, which are ultimately some of the current trends in Physical Education because these features help to achieve a range of educationally beneficial objectives related to many different domains, such as the physical, lifestyle, affective, social and cognitive (Dyson et al., 2004; Kirk, 2010, 2013). All in all, the use of CLIL in the area of Physical Education may help align the subject with its current principles (Lamb and King, 2019).

Participants also stated that, according to their experiences, introducing CLIL contributed to the development of positive relationships between students (Casal, 2016; Bower, 2019). Similarly, a case study analyzing CLIL with Physical Education concluded that there was an improvement in social interaction and a sense of sharing among students (Christopher et al., 2012). Therefore, using CLIL could entail a change in the social dynamics of the group (Salvador-García et al., 2018). However, our quantitative findings do not totally align with the interview findings, since no significant differences were found when comparing pre- and post-test measures of the sociometric questionnaire. Despite these results, in general terms, there are some interesting outcomes when comparing the results obtained by gender. In this regard, three of the questions show opposing trends when the mean scores of males and females are compared. This might be related to the differences by gender that are often found regarding both interpersonal relations, for example, focusing on acceptance within the group (Andueza and Lavega, 2017), and CLIL (Doiz et al., 2014; Fernandez-Barrionuevo and Baena-Extremera, 2018). Therefore, it is necessary to carry out further research on this topic, because the effects on sociometric measures might need longer to appear (van der Wilt et al., 2019).

Regarding the first and second purposes of Physical Education (Hardman, 2011), that is to say, those closely linked to the physical sphere, interviewees also expressed their opinions on CLIL’s possible effects and accelerometry allowed us to analyze it objectively. Contrary to the stereotypes of many critical voices who claim that physical activity levels decrease in Physical Education with CLIL (Coral et al., 2017; Martinez and Garcia, 2017), we did find evidence in both the interviews and accelerometry results that the quality of physical activity is not necessarily diminished. In addition, despite some teachers’ concerns about their explanations being longer when applying CLIL (Lo and Macaro, 2015; Salvador-García and Chiva-Bartoll, 2017), the percentage of time that students were engaged in MVPA was also maintained.

We can explain such physical activity analytical results only by combining them with the findings from the qualitative study. In our case, the interviewed students were willing to help each other understand, maybe due to the high levels of motivation that are usually linked to Physical Education through CLIL (Baena-Extremera et al., 2017; Lamb and King, 2019). In addition, using CLIL may entail an increase in students’ attentiveness (Zindler, 2013; Salvador-García and Chiva-Bartoll, 2017), or the use of teaching strategies that teachers employ to enhance students’ understanding (Gomez and Jimenez-Silva, 2012; Salvador-García et al., 2019). In this regard, teachers who apply CLIL tend to be more concerned with the vocabulary and language structures they use to ensure effective communication (Ting, 2011; Zindler, 2013). However, this contrasts with other authors who claim that some teachers may overuse language learning materials such as flashcards and, consequently, the teacher talking time is increased while students’ activity time is diminished (Coral et al., 2017). More research is needed to further investigate these aspects and better understand how Physical Education sessions are carried out when the CLIL approach is used.

## Conclusion

Addressing the stated research objective through the application of a unique mixed method strategy with exploratory sequential design has been useful in our study, as it puts students and teachers at the center of CLIL research in the field of Physical Education and focuses on the basic features of Physical Education. One of the main purposes of Physical Education refers to personal, social and moral development (Hardman, 2011). In this regard, the interviews undertaken revealed the potential role that CLIL may play in improving social relationships between students; however, the sociometric questionnaire findings only show some very slight trends in gender-based outcomes. In any case, the interpretation of these results yields some interesting inferences leading to the conclusion that the use of a CLIL approach might change the social interactions in class for the better. Thus, CLIL might strengthen one of the main purposes of Physical Education.

Regarding the aims of Physical Education related to development of motor and sport-specific skills and promotion of health-related fitness and active lifestyles (Hardman, 2011), some participants wondered whether physical activity levels would be altered due to the application of CLIL. Despite being recurrent in their responses, the interviewees did not reach any consensus about this concern, so in the second phase of the study a measurement of MVPA through accelerometry objectively showed that MVPA was not jeopardized, with the CLIL group obtaining higher levels of MVPA than the non-CLIL group. These results represent an outstanding finding, since they certainly shed new light on one of the most widespread concerns in this field. Therefore, according to our study, the CLIL approach does not necessarily hinder the basic purposes of Physical Education, which was a concern for many scholars (Merino, 2016; Coral et al., 2017; Pérez-Cañado, 2017). In fact, it might contribute to its development.

This study has some limitations. The quantitative part of the study relies on data collected with a limited sample. Also, given the fact that the study was carried out in Spain, the findings might not be transferable to all contexts. However, these are common issues that come with educational research that attempts to uncover intervention programs (Sandoval and Bell, 2004). We are convinced, instead, that by highlighting the views of these groups of participants and sharing the quantitative measures obtained, we have contributed to knowledge on how the CLIL approach can influence Physical Education. Furthermore, it should be noted that qualitative research does not attempt to generalize knowledge in a categorical way, but rather aspires to deepen our knowledge about contextualized phenomena. It does take advantage of the results obtained to relate them to existing literature and thus increase the conceptual framework of the field (Denzin and Lincoln, 2005). It is in this way that, from an interpretative paradigm, this study helps broaden knowledge in Physical Education research (Pérez-Samaniego et al., 2011).

## Data Availability Statement

The datasets generated for this study are available on request to the corresponding author.

## Ethics Statement

This studies involving human participants were reviewed and approved by Deontological Commission of the University Jaume I:UJI-PREDOC/2016/03. Written informed consent to participate in this study was provided by the participants’ legal guardian/next of kin.

## Author Contributions

OC-B and CS-G conceived the idea presented and designed the study. CS-G and CC-P organized the database. PR-M and CC-P performed the statistical analysis. CS-G wrote the first draft of the manuscript. OC-B, CS-G, and CC-P reviewed the methodological sections of the manuscript. All authors contributed to revision of the manuscript and read and approved the submitted version.

## Conflict of Interest

The authors declare that the research was conducted in the absence of any commercial or financial relationships that could be construed as a potential conflict of interest.
